# Association between weight-adjusted waist index and risk of mortality and disease progression in participants with chronic kidney disease: a prospective study from the UK Biobank

**DOI:** 10.3389/fnut.2026.1646414

**Published:** 2026-02-25

**Authors:** Chengkai Wu, Chuqing Pan, Li Liu, Wenyuan Li

**Affiliations:** 1 School of Public Health, Southern Medical University, Guangzhou, China; 2 School of Health Management, Southern Medical University, Guangzhou, China; 3 Nanfang Hospital, Southern Medical University, Guangzhou, China; 4 Department of Health Management, Nanfang Hospital, Southern Medical University, Guangzhou, China

**Keywords:** chronic kidney disease, end-stage kidney disease, mortality, UK Biobank, weight-adjusted waist index

## Abstract

**Background:**

The weight-adjusted waist index (WWI), a novel metric reflecting central obesity, has shown potential as a predictor of adverse health outcomes. This study investigated the associations between WWI and all-cause mortality, cardiovascular disease (CVD) mortality, and the incidence of end-stage kidney disease (ESKD) among participants with chronic kidney disease (CKD), and further compared its predictive performance with that of conventional obesity indices.

**Methods:**

This prospective cohort study was conducted with data from the United Kingdom Biobank (UKB). Participants with CKD at baseline were included, while those with ESKD, missing data on WWI or other obesity indicators, or incomplete covariate information were excluded. Follow-up time was calculated from the baseline to the occurrence of disease diagnoses, surgical interventions, mortality or the censoring date (31 December 2021). CKD was diagnosed using baseline clinical data, and WWI was calculated as the waist circumference (WC, cm) divided by the square root of body weight (kg). Cox proportional hazards models and restricted cubic spline (RCS) analysis were employed to examine the associations between WWI and all outcomes. Receiver operating characteristic (ROC) curve analysis were conducted to compare the predictive performance of WWI, body mass index (BMI) and WC.

**Results:**

During a follow-up period of 269,858.2 person-years for 22,523 participants, 3,874 all-cause deaths, 786 CVD deaths and 823 ESKD events were recorded. WWI exhibited non-linear association with all-cause mortality and ESKD incidence (*P* for non-linear < 0.001), and linear association with CVD mortality (*P* for non-linear = 0.175). WWI values above the threshold (10.5 cm/√kg) showed significant positive associations with all outcomes (all *P* value < 0.001). WWI demonstrated superior overall predictive performance compared with traditional obesity indicators, particularly in predicting all-cause mortality.

**Conclusion:**

These findings suggest that an elevated WWI is associated with an increased risk of all-cause mortality, CVD mortality and ESKD in patients with CKD, particularly in high WWI groups. Although WWI demonstrated statistically superior discriminatory performance compared with BMI and WC, its overall discriminative capacity remained modest. Accordingly, WWI may serve as a complementary anthropometric indicator for risk assessment in CKD, further studies are required to establish its clinical applicability.

## 1 Introduction

Chronic kidney disease (CKD) is a significant health burden, affecting approximately 13% of the global population and projected to impact 2.63 million individuals worldwide by 2030 (1). CKD is associated with a significantly higher risk of mortality compared to those with normal renal function (2). According to the Global Burden of Disease Study published in 2017, CKD was responsible for 1.2 million deaths globally, which increased to 1.42 million in 2019, alongside a considerable number of cardiovascular-related deaths attributed to declining kidney function (1, 3). Between 1990 and 2019, CKD rose from 19*^th^* to 11*^th^* leading cause of death, and it is predicted to rank among the top five causes of mortality by 2040 (4). Hence, an accurate identification of factors influencing CKD progression and prognosis is critical for enhancing patient survival.

Obesity, a multifactorial metabolic disorder and a major global health concern, has been on the rise since the 1980s, with nearly one-third of the global population being classified as obese (5, 6). Chronic obesity is strongly associated with an increased risk of cardiovascular diseases (CVD), metabolic disorders, certain types of cancer, and significantly increasing mortality rates (7–11). Obesity has been identified as a driver of CKD progression, with complex underlying mechanisms, including hemodynamic changes, inflammation, oxidative stress, and activation of the renin-angiotensin-aldosterone system (12). It has also been correlated with CKD in metabolically healthy individuals with obesity, both in the general population and in specific disease cohorts (13–15).

Commonly used clinical indicators of obesity, such as body mass index (BMI) and waist circumference (WC). Although BMI is a widely used predictor of premature mortality, it fails to differentiate between adipose tissue and lean body mass or between central and peripheral fat distribution, thereby presenting inherent limitations. Emerging evidence suggests that body composition and fat distribution are precise predictors of adverse health outcomes. WC is a well-recognized marker of abdominal obesity. However, its strong correlation with BMI restricts its use as an independent measure. In 2018, Park et al. introduced the weight-adjusted waist index (WWI), an anthropometric indicator of central obesity that considers both muscle and fat mass, preserving the advantages of WC while mitigating its correlation with BMI (16). Hence, WWI is potentially more applicable in obesity-related research and clinical practice (17). The prevalence of newly diagnosed hypertension, diabetes, CKD, all-cause mortality, and CVD mortality are associated with WWI (18–22). Similar results were found in certain disease populations, such as type 2 diabetes and liver disease (23–25). Li et al. demonstrated that WWI is positively associated with albuminuria, with better predictive ability than other obesity indicators (26).

To the best of our knowledge, no prior research has specifically explored the association between WWI and adverse outcomes, including all-cause mortality, CVD mortality and end-stage renal disease (ESKD), in patients with CKD. Accordingly, this study aims to assess the association between WWI and these critical outcomes in patients with CKD utilizing data from the United Kingdom Biobank (UKB).

## 2 Materials and methods

### 2.1 Study design and participants

**FIGURE 1:**
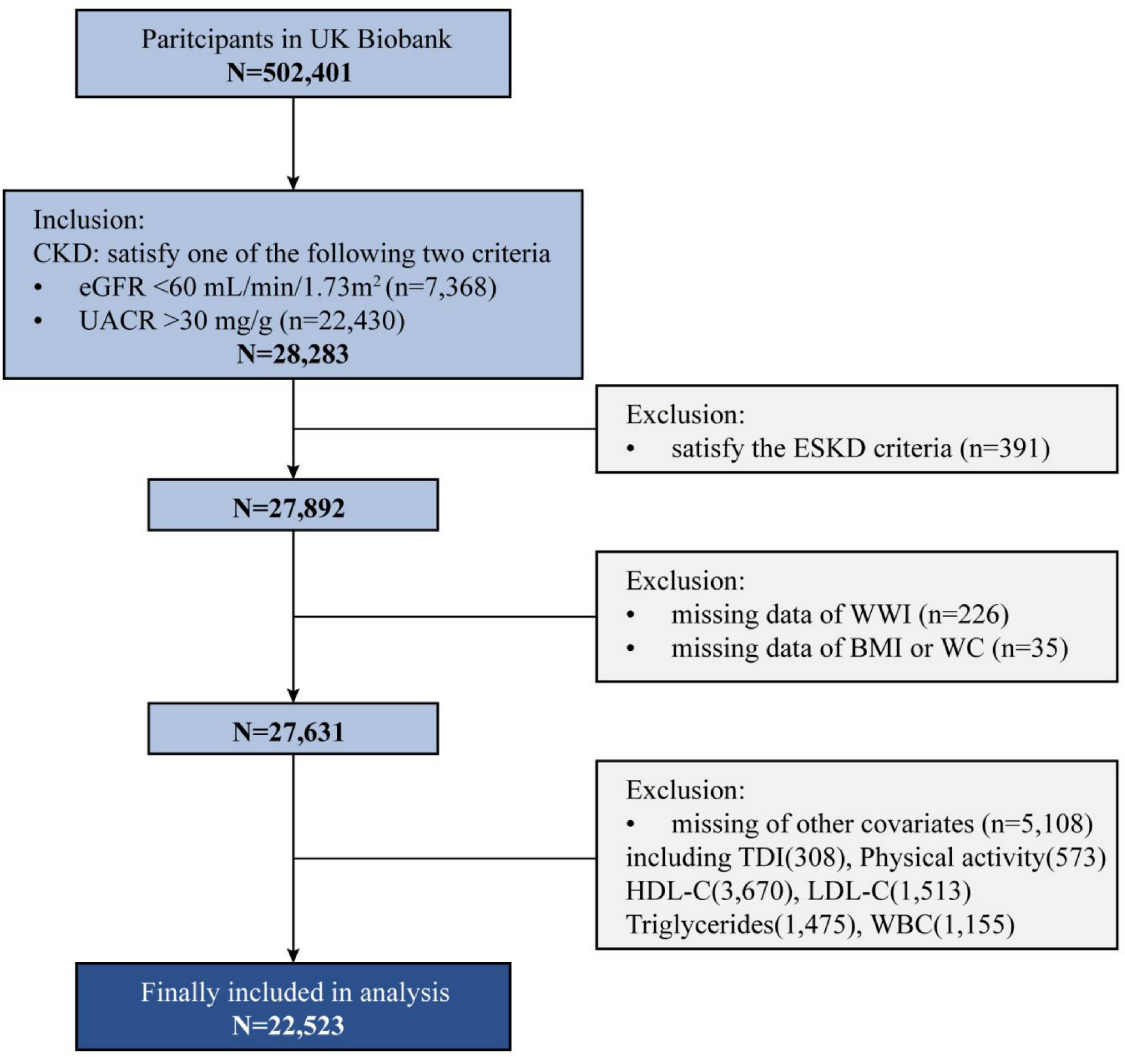
Participant selection flowchart. WWI, weight-adjusted waist index; eGFR, estimated glomerular filtration rate; UACR, urinary albumin-to-creatinine ratio; ESKD, end-stage kidney disease; BMI, body mass index; WC, waist circumference; TDI, townsend deprivation index; HDL-C, high-density lipoprotein cholesterol; LDL-C, low-density lipoprotein cholesterol; WBC, white blood cell count.

This prospective population-based cohort study included participants enrolled in the UKB, a large cohort study that recruited almost half a million participants aged 40–69 years in the UK between 2006 and 2010 (5% response rate) through postal invitation. The UKB was approved by the North West Multicenter Research Ethics Service (NHS National Research Ethics Service, 16/NW/0274). Written informed consent was obtained from all participants for data collection, analysis, and linkage. Each participant completed a touchscreen questionnaire and a nurse-led interview and underwent physical measurements in one of the 22 assessment centers across England, Scotland, and Wales. Blood and urine samples were collected for biological marker assessments. This study was conducted under approved application number 532248. The inclusion criteria required participants to meet the definition of CKD at baseline (*n* = 28,283). The exclusion criteria included meeting the definition of ESKD at baseline (*n* = 391), having missing data on the WWI (*n* = 226) or other obesity-related indicators (*n* = 35), and lacking complete information on covariates (*n* = 5,108). After applying these criterias, a total of 22,523 participants were retained for the final analysis (Figure 1). This study followed the Strengthening the Reporting of Observational Studies in Epidemiology (STROBE) reporting guideline for cohort studies.

### 2.2 Assessment of CKD

Chronic kidney disease was identified based on baseline biological sample testing. In accordance with the “Kidney Disease: Improving Global Outcomes (KDIGO) 2021 Guidelines,” CKD is defined by a urinary albumin-to-creatinine ratio (UACR) exceeding 30 mg/g and/or an estimated glomerular filtration rate (eGFR) below 60 mL/min/1.73 m^2^ (4). The eGFR was calculated using the Chronic Kidney Disease Epidemiology Collaboration (CKD-EPI) 2021 creatinine-based equation for standardized creatinine (27).

### 2.3 Assessment of WWI, BMI, and WC

This study utilized baseline anthropometric data from the UKB, which were collected by trained personnel under standardized protocols with regularly calibrated equipment to ensure high measurement accuracy. Body weight (kg) was assessed using the Tanita BC-418 MA body composition analyzer after the participants removed their shoes and heavy outer clothing and standing height (m) was measured without shoes using a Seca 202 (28). WC was measured to the nearest 0.1 cm at the level of the umbilicus using a Wessex non-stretchable spring tape, with the participant in a resting standing position. BMI was calculated as body weight divided by the square of standing height. WWI (cm/√kg) was calculated as WC divided by the square root of body weight and rounded to two decimal places.

### 2.4 Definitions of outcomes

The outcome events of this study included all-cause mortality, CVD mortality and ESKD incidence. Mortality data were ascertained through data linkage with national death registries (NHS Information Center for participants in England and Wales and NHS Central Registry for participants in Scotland). CVD mortality was determined based on the primary cause of death, identified using ICD-10 codes (I20-I26, I60-I69). ESKD was defined as CKD stage G5 or the requirement for renal replacement therapy, including long-term dialysis or kidney transplantation, identified through hospital admission ICD-10 codes and OPCS-4 codes or ICD-10 codes recorded in any position of a death certificate (Supplementary Table 1).

### 2.5 Covariates

This study included multiple potential covariates, including age, sex, education level (college/above, high school/equivalent, less than high school or other), townsend deprivation index (TDI), annual household income (< £18 000, £18 000 to £51 999, ≥ £52 000 or other), smoking status (non-smoker, ex-smoker, current smoker or other), alcohol consumption (never, light, excessive or other), healthy diet score (poor, medium or ideal), physical activity (goal or below goal), white blood cell (WBC) count, low-density lipoprotein cholesterol (LDL-C), high-density lipoprotein cholesterol (HDL-C), triglyceride, eGFR, UACR, hypertension (yes or no), diabetes mellitus (yes or no) and hyperuricemia (yes or no), which were collected through baseline questionnaires, verbal interviews, and laboratory test indicators. The TDI was calculated using national census data and assigned postal codes of residence and categorized by quartiles, with a lower score indicating a higher area level of socioeconomic status (29). Physical activity was defined according to the American Heart Association (AHA) recommendations, with goal physical activity defined as engaging in ≥150 min/week of moderate-intensity activity, ≥ 5 min/week of vigorous-intensity activity or an equivalent combination (30). Healthy diet score were determined based on a touchscreen food frequency questionnaire (FFQ), including the dietary consumption of poultry, beef, lamb/mutton, processed meat, oily fish, non-oily fish, fresh fruit, dried fruit, raw vegetables and cooked vegetables and categorized into poor, medium or ideal groups following a previous UKB study (31). Hypertension was identified by self-reported physician diagnosis, use of antihypertensive medication, or elevated baseline blood pressure (systolic ≥ 140 mmHg or diastolic ≥ 90 mmHg). Diabetes mellitus was identified by self-reported diagnosis, use of glucose-lowering medication, or increased fasting glucose (≥7.0 mmol/L), 2-h plasma glucose (≥11.1 mmol/L), or HbA1c (≥6.5%). Hyperuricemia was identified by increased serum uric acid levels (>7.0 mg/dL in men and > 6.0 mg/dL in women). Standardized protocols for the collection and processing of baseline blood and urine samples have been previously described (32). Detailed definitions and classification criteria for covariates are provided in the Supplementary Methods.

### 2.6 Statistical analysis

Continuous variables with a non-normal distribution were expressed as median (interquartile range, IQR), whereas those with a normal distribution were expressed as mean (standard deviation, SD). Categorical variables were presented as counts (percentages). Group comparisons were performed using the Kruskal–Wallis test or one-way ANOVA for continuous variables, and the χ^2^ test for categorical variables. The association of WWI, BMI and WC with outcomes was analyzed using the Cox proportional hazards model and expressed as hazard ratios (HRs) and 95% confidence intervals (CIs). In the analysis, WWI, BMI and WC were included in the model as continuous variables and quartiles, respectively. Model 1 was adjusted for age and sex. Model 2 further included annual household income, smoking status, alcohol consumption, education, TDI, healthy diet score, physical activity, hyperuricemia, hypertension, diabetes mellitus, HDL-C, LDL-C, triglycerides and WBC count. Model 3 additionally adjusted for eGFR and UACR. Survival time was defined as the duration from the baseline to the outcome event or censoring date (31 December 2021). Kaplan–Meier survival curves for the different groups were compared using log-rank tests. Restricted cubic spline (RCS) regression was used to examine the non-linear association between WWI and outcomes, and a two-stage Cox proportional hazards regression model was constructed based on the threshold point. The effectiveness of WWI, BMI, and WC in predicting outcomes was evaluated using smoothed receiver operating characteristic (ROC) curves. Pairwise comparisons of the areas under the ROC curves (AUCs) were performed using a non-parametric bootstrap procedure with 2,000 iterations to assess statistical differences between indices. Bonferroni correction was applied to adjust for multiple comparisons, and corresponding *p*-values were reported to determine the significance of AUC differences.

To ensure the robustness of our findings, three different sensitivity analysis were performed. The first excluded participants with major CVDs (myocardial infarction, coronary heart disease, or stroke) or cancer at baseline; the second excluded participants with less than 2 years of follow-up; and the third employed Fine–Gray competing risk models to account for potential competing events, where death was considered a competing risk for ESKD incidence and non-CVD death for CVD mortality. Additional subgroup analysis and interaction tests evaluated the association between WWI and outcomes across sex, age and disease status.

All analysis were performed using R software version 4.4.1 [R Core Team (33). R: A language and environment for statistical computing. R Foundation for Statistical Computing, Vienna, Austria. URL^1^ ]. Statistical significance was defined as a two-tailed *p*-value of <0.05.

## 3 Results

### 3.1 Baseline characteristics

This study included 22,523 participants who met the eligibility criteria. The median (IQR) age of the participants was 61 (54–66) years, and 9,289 (52.9%) were female. The median follow-up duration was 12.6 (11.7–13.4) years. During 269,858.2 person-years of follow-up, 3,874 mortality occurred, including 786 cardiovascular mortality, and 823 ESKD incidence. The median WWI was 10.5. Participants were grouped according to WWI quartiles: quartile 1 (6.59–9.96), quartile 2 (9.96–10.50), quartile 3 (10.50–11.10) and quartile 4 (11.10–14.91). Participants with a higher WWI were older, more likely to be male, had a higher TDI, lower annual household income, below goal physical activity, unhealthy diet and a higher prevalence of diabetes, hypertension and hyperuricemia (Table 1).

**TABLE 1:** Baseline characteristics of study participants according to weight-adjusted waist index.

Characteristic	Total (*N* = 22, 523)	Participants, No. (%)^a^				
		Quartile 1 (*n* = 5, 593)	Quartile 2 (*n* = 5, 640)	Quartile 3 (*n* = 5, 656)	Quartile 4 (*n* = 5, 634)	*P*-value
Age, median (IQR), y	61 (54–66)	59 (50–64)	61 (54–65)	62 (56–66)	63 (58–67)	<0.001
**Sex**						
Female	11,477 (51.0)	4,319 (77.2)	2,834 (50.2)	2,267 (40.1)	2,057 (36.5)	<0.001
Male	11,046 (49.0)	1,274 (22.8)	2,806 (49.8)	3,389 (59.9)	3,577 (63.5)	
**TDI, quartile**						
Quartile 1	5,746 (25.5)	1,648 (29.5)	1,546 (27.4)	1,447 (25.6)	1,105 (19.6)	<0.001
Quartile 2	5,617 (24.9)	1,542 (27.6)	1,435 (25.4)	1,393 (24.6)	1,247 (22.1)	
Quartile 3	5,633 (25.0)	1,400 (25.0)	1,375 (24.4)	1,398 (24.7)	1,460 (25.9)	
Quartile 4	5,527 (24.5)	1,003 (17.9)	1,284 (22.8)	1,418 (25.1)	1,822 (32.3)	
**Education level**						
College/above	5,883 (26.1)	1,908 (34.1)	1,555 (27.6)	1,357 (24.0)	1,063 (18.9)	<0.001
High school/equivalent	6,733 (29.9)	1,924 (34.4)	1,711 (30.3)	1,616 (28.6)	1,482 (26.3)	
Less than high school	3,902 (17.3)	814 (14.6)	1,034 (18.3)	1,065 (18.8)	989 (17.6)	
Other^b^	6,005 (26.7)	947 (16.9)	1,340 (23.8)	1,618 (28.6)	2,100 (37.3)	
**Annual household income, £**						
<18,000	6,204 (27.5)	1,158 (20.7)	1,407 (24.9)	1,613 (28.5)	2,026 (36.0)	<0.001
18,000–51,999	9,109 (40.4)	2,361 (42.2)	2,418 (42.9)	2,314 (40.9)	2,016 (35.8)	
>52,000	3,209 (14.2)	1,156 (20.7)	864 (15.3)	726 (12.8)	463 (8.2)	
Other^c^	4,001 (17.8)	918 (16.4)	951 (16.9)	1,003 (17.7)	1,129 (20.0)	
**Smoking status**						
Non-smoker	10,858 (48.2)	3,278 (58.6)	2,806 (49.8)	2,525 (44.6)	2,249 (39.9)	<0.001
Ex-smoker	8,696 (38.6)	1,689 (30.2)	2,123 (37.6)	2,348 (41.5)	2,536 (45.0)	
Current smoker	2,796 (12.4)	599 (10.7)	681 (12.1)	735 (13.0)	781 (13.9)	
Other^d^	173 (0.8)	27 (0.5)	30 (0.5)	48 (0.8)	68 (1.2)	
**Alcohol intake**						
Never	5,972 (26.5)	1,284 (23.0)	1,305 (23.1)	1,483 (26.2)	1,900 (33.7)	<0.001
Light	7,831 (34.8)	2,077 (37.1)	2,004 (35.5)	1,903 (33.6)	1,847 (32.8)	
Excessive	8,633 (38.3)	2,218 (39.7)	2,313 (41.0)	2,251 (39.8)	1,851 (32.9)	
Other^d^	87 (0.4)	14 (0.3)	18 (0.3)	19 (0.3)	36 (0.6)	
**Physical activity**						
Below goal	12,567 (55.8)	2,730 (48.8)	2,947 (52.3)	3,220 (56.9)	3,670 (65.1)	<0.001
Goal	9,956 (44.2)	2,863 (51.2)	2,693 (47.7)	2,436 (43.1)	1,964 (34.9)	
**Healthy diet score**						
Poor	12,134 (53.9)	2,530 (45.2)	2,961 (52.5)	3,223 (57.0)	3,420 (60.7)	<0.001
Medium	6,337 (28.1)	1,767 (31.6)	1,600 (28.4)	1,491 (26.4)	1,479 (26.3)	
Ideal	4,052 (18.0)	1,296 (23.2)	1,079 (19.1)	942 (16.7)	735 (13.0)	
**Diabetes mellitus**						
No	15,710 (69.8)	4,634 (82.9)	4,203 (74.5)	3,779 (66.8)	3,094 (54.9)	<0.001
Yes	6,813 (30.2)	959 (17.1)	1,437 (25.5)	1,877 (33.2)	2,540 (45.1)	
**Hypertension**						
No	4,565 (20.3)	1,757 (31.4)	1,121 (19.9)	890 (15.7)	797 (14.1)	<0.001
Yes	17,958 (79.7)	3,836 (68.6)	4,519 (80.1)	4,766 (84.3)	4,837 (85.9)	
**Hyperuricemia**						
No	15,741 (69.9)	4,683 (83.7)	4,033 (71.5)	3,676 (65.0)	3,349 (59.4)	<0.001
Yes	6,782 (30.1)	910 (16.3)	1,607 (28.5)	1,980 (35.0)	2,285 (40.6)	
HDLC, mean (SD), mmol/L	1.31 (0.52)	1.57 (0.54)	1.34 (0.49)	1.24 (0.44)	1.15 (0.40)	<0.001
LDLC, mean (SD), mmol/L	3.34 (1.30)	3.44 (1.17)	3.46 (1.32)	3.32 (1.34)	3.10 (1.31)	<0.001
Triglycerides, mean (SD), mmol/L	1.67 (1.29)	1.23 (0.83)	1.68 (1.21)	1.88 (1.31)	2.06 (1.42)	<0.001
WBC, mean (SD), × 10^9^/L	7.22 (2.52)	6.69 (2.35)	7.10 (2.35)	7.40 (2.50)	7.79 (2.55)	<0.001
**BMI, quartile**						
Quartile 1	5,373 (23.9)	2,972 (53.1)	1,292 (22.9)	744 (13.2)	365 (6.5)	< 0.001
Quartile 2	5,659 (25.1)	1,484 (26.5)	1,857 (32.9)	1,447 (25.6)	871 (15.5)	
Quartile 3	5,687 (25.2)	765 (13.7)	1,534 (27.2)	1,816 (32.1)	1,572 (27.9)	
Quartile 4	5,804 (25.8)	372 (6.7)	957 (17.0)	1,649 (29.2)	2,826 (50.2)	
**WC, quartile**						
Quartile 1	5,338 (23.7)	3,952 (70.7)	1,076 (19.1)	264 (4.7)	46 (0.8)	<0.001
Quartile 2	5,730 (25.4)	1,293 (23.1)	2,475 (43.9)	1,481 (26.2)	481 (8.5)	
Quartile 3	5,595 (24.8)	301 (5.4)	1,610 (28.5)	2,254 (39.9)	1,430 (25.4)	
Quartile 4	5,860 (26.0)	47 (0.8)	479 (8.5)	1,657 (29.3)	3,677 (65.3)	

TDI, townsend deprivation index; HDLC, high-density lipoprotein cholesterol; LDLC, low-density lipoprotein cholesterol; WBC, white blood cell count; BMI, body mass index; WC, waist circumference.

*^a^*Due to rounding, percentages may not add up to exactly 100%.

*^b^*Other includes none of the above, prefer not to answer or missing data.

*^c^*Other includes do not know, prefer not to answer or missing data.

*^d^*Other includes prefer not to answer or missing data.

### 3.2 Association between WWI with all-cause mortality, CVD mortality and ESKD

The KM survival analysis showed significant differences in all-cause (Supplementary Figure 1A), CVD (Supplementary Figure 1B) mortality and ESKD incidence (Supplementary Figure 1C) between the different WWI quartiles. A higher WWI was associated with higher all-cause and CVD mortality during follow-up (*p* < 0.0001 for all log-rank tests).

Based on COX regression analysis, continues WWI was associated with outcomes in all models. The results in model3 showed significant positive associations with all-cause mortality (per unit increase; HR: 1.30, 95% CI: 1.24–1.37), CVD mortality (1.26, 1.12–1.41) and ESKD incidence (1.27, 1.14–1.43) (Table 2). Additionally, compared with those in quartile 1, participants in quartile 4 had a significantly higher risk of all-cause mortality (HR: 1.44, 95% CI: 1.28–1.63), cardiovascular mortality (1.42, 1.06–1.90) and ESKD incidence (1.41, 1.09–1.82). In model 3, participants in quartile 4 of BMI and WC were not significantly associated with all-cause and CVD mortality compared to quartile 1. Highest levels of BMI was found to be protective against the ESKD incidence (Supplementary Tables 2, 3).

**TABLE 2:** Association of WWI with all-cause mortality, CVD mortality and ESKD incidence.

WWI	Cases/*N*	Hazard ratio (95% CI)^a^		
		Model 1	Model 2	Model 3
**All-cause mortality**				
WWI (Per-1 increase)	3,874/22,523	1.47 (1.41–1.53)***	1.27 (1.21–1.33)***	1.30 (1.24–1.37)***
**WWI (quartile)**				
Quartile 1	534/5,593	1 (ref)	1 (ref)	1 (ref)
Quartile 2	780/5,640	1.13 (1.01–1.26)*	1.01 (0.90–1.13)	1.05 (0.93–1.19)
Quartile 3	1,007/5,656	1.30 (1.17–1.45)***	1.06 (0.94–1.18)	1.11 (0.98–1.26)
Quartile 4	1,553/5,634	1.98 (1.79–2.20)***	1.37 (1.23–1.53)***	1.44 (1.28–1.63)***
*P* for trend		<0.001	<0.001	<0.001
**CVD cause mortality**				
WWI (Per-1 increase)	407/22,523	1.70 (1.55–1.88)***	1.26 (1.13–1.40)***	1.26 (1.12–1.41)***
**WWI (quartile)**				
Quartile 1	77/5,593	1 (ref)	1 (ref)	1 (ref)
Quartile 2	140/5,640	1.22 (0.92–1.62)	0.98 (0.74–1.30)	1.02 (0.75–1.38)
Quartile 3	206/5,656	1.55 (1.18–2.03)**	1.04 (0.79–1.37)	1.05 (0.78–1.41)
Quartile 4	363/5,634	2.66 (2.06–3.44)***	1.39 (1.06–1.82)*	1.42 (1.06–1.90)*
*P* for trend		<0.001	<0.001	<0.001
**ESKD incidence**				
WWI (Per-1 increase)	559/22,523	1.52 (1.38–1.66)***	1.12 (1.01–1.24)*	1.27 (1.14–1.43)***
**WWI (quartile)**				
Quartile 1	127/5,593	1 (ref)	1 (ref)	1 (ref)
Quartile 2	166/5,640	1.07 (0.84–1.35)	0.82 (0.65–1.05)	1.01 (0.78–1.31)
Quartile 3	205/5,656	1.25 (0.99–1.57)	0.82 (0.64–1.04)	1.01 (0.78–1.31)
Quartile 4	325/5,634	2.04 (1.64–2.53)***	1.06 (0.83–1.33)	1.41 (1.09–1.82)**
*P* for trend		<0.001	>0.05	<0.01

WWI, weight-adjusted waist index; CVD, cardiovascular; ESKD, end-stage kidney disease.

*^a^*Model 1: age and sex were adjusted. Model 2: age, sex, annual household income, smoking status, alcohol intake, education level, townsend deprivation index, healthy diet score, physical activity, hyperuricemia, hypertension, diabetes mellitus, HDLC, LDLC, Triglycerides, and WBC were adjusted. Model 3: age, sex, annual household income, smoking status, alcohol intake, education level, townsend deprivation index, healthy diet score, physical activity, hyperuricemia, hypertension, diabetes mellitus, HDLC, LDLC, Triglycerides, WBC, eGFR, and UACR were adjusted.

*P-value < 0.05;

**P-value < 0.01;

***P-value < 0.001.

**FIGURE 2:**
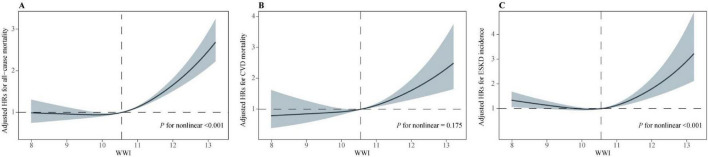
Restricted cubic spline (RCS) analysis of WWI with all outcomes. **(A)** all-cause mortality. **(B)** CVD mortality. **(C)** ESKD incidence. WWI, weight-adjusted waist index; CVD, cardiovascular disease; ESKD, end-stage kidney disease.

Restricted cubic spline analysis showed that WWI was non-linearly associated with all-cause mortality (*p* for non-linear < 0.001) and ESKD incidence (*p* for non-linear < 0.001), while it had a linear association with CVD mortality (*p* for non-linear = 0.175) (Figure 2). Notably, all RCS curves exhibited a consistent inflection pattern, with breakpoints located approximately at the median WWI value (10.5); therefore, the median was selected as the threshold for consenquent analysis. Cox regression analysis showed a positive correlation between WWI and all-cause mortality (HR: 1.46, 95% CI: 1.34–1.58), CVD mortality (1.34, 1.13–1.58) and ESKD incidence (1.55, 1.29–1.86) to the right of the threshold (Table 3), whereas to the left, there was no significant correlation. The RCS analysis of BMI (Supplementary Figure 2) and WC (Supplementary Figure 3) demonstrated relatively consistent patterns. A significant non-linear U-shaped association was observed for both all-cause mortality and ESKD incidence, with significance evident on either side of the breakpoint. In contrast, the association with CVD mortality exhibited a linear trend.

**TABLE 3:** Threshold effect analysis of WWI.

WWI	N	All-cause mortality		CVD mortality		ESKD incidence	
		Cases	HR (95% CI)	Cases	HR (95% CI)	Cases	HR (95% CI)
<10.5	10,664	1,220	1.04 (0.90–1.20)	202	1.03 (0.74–1.43)	279	1.07 (0.81–1.41)
≥10.5	11,859	2,654	1.46 (1.34–1.58)***	584	1.34 (1.13–1.58)***	544	1.55 (1.29–1.86)***

WWI, weight-adjusted waist index; CVD, cardiovascular disease; ESKD, end-stage kidney disease.

***P-value < 0.001.

### 3.3 Subgroup analysis and sensitivity analysis

Subgroup analysis and interaction tests stratified by age, sex, and disease status showed a significant interaction between WWI and CVD mortality according to sex, hypertension, and hyperuricemia (*p* < 0.05) (Supplementary Table 4). However, the direction of this association remained consistent across the subgroups. The association between WWI and ESKD incidence remained consistent across age, sex, and other disease subgroups, except in the hyperuricemia group (*p* < 0.05). In sensitivity analysis, the associations of WWI with all-cause and CVD mortality were largely unchanged. Regarding ESKD incidence, the results were consistent across analysis except for the second sensitivity analysis, in which a slight deviation was observed (Supplementary Tables 5–7).

### 3.4 Predictive performance of WWI, BMI, and WC

**FIGURE 3:**
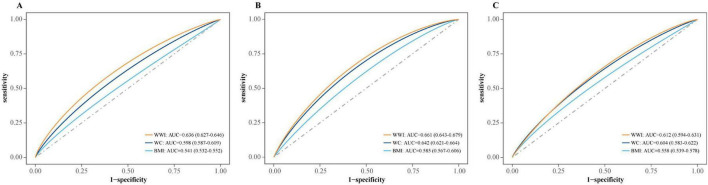
The receiver operating characteristics (ROC) analysis of WWI, BMI, and WC on all outcomes. **(A)** All-cause mortality. **(B)** CVD mortality. **(C)** ESKD incidence. WWI, weight-adjusted waist index; CVD, cardiovascular disease; ESKD, end-stage kidney disease; AUC, the area under the curve; BMI, body mass index; WC, waist circumference.

Figure 3 demonstrates the predictive performance of WWI, WC and BMI for all-cause (Figure 2A) and CVD (Figure 2B) mortality and ESKD incidence (Figure 2C). The area under the curves (AUCs) for predicting all-cause mortality were 0.636, 0.598, and 0.541, respectively. The CVD mortality rates were 0.661, 0.642, and 0.585, ESKD incidences were 0.612, 0.604 and 0.558, respectively. Compared with other traditional obesity indicators, WWI demonstrated a significantly higher AUC for predicting all-cause mortality (*p* < 0.0001 for all AUC comparisons). It also outperformed BMI in predicting cardiovascular mortality and ESKD, while showing comparable performance to WC (Supplementary Table 8).

## 4 Discussion

In this prospective cohort study based on participants with CKD from the UK Biobank, the associations of the WWI with all-cause mortality, CVD mortality and ESKD incidence had been comprehensively evaluated. Higher WWI levels were consistently associated with increased risks of all-cause, cardiovascular mortality and ESKD incidence. RCS analysis further revealed a non-linear association of WWI with all-cause mortality and ESKD incidence, and a linear association with CVD mortality, with risk elevations evident above the threshold 10.5 cm/√kg. These associations remained robust across extensive subgroup and sensitivity analysis. Moreover, compared with BMI and WC, WWI exhibited superior predictive performance for all-cause mortality and showed better predictive ability than BMI for cardiovascular mortality and ESKD.

Previous research has demonstrated that BMI and WC, the most commonly used anthropometric indicators in clinical settings, were associated with the incidence of CKD (34). In the fully adjusted model of this study, higher BMI and WC were not significantly associated with all-cause and CVD mortality, while an inverse association was observed with the incidence of ESKD. These findings are consistent with the well-documented “obesity paradox” in CKD populations, whereby higher anthropometric measures are paradoxically linked to lower mortality risk (35). Several factors may contribute to this phenomenon. First, BMI and WC are limited in their ability to accurately reflect body fat distribution and composition, thereby weakening their predictive capacity for obesity-related outcomes. Second, elevated BMI and WC may serve as proxies for better nutritional reserves, which could mitigate the adverse effects of obesity in CKD patients. These results align with previous studies and further emphasize the limited prognostic utility of BMI and WC in this population (36).

Weight-adjusted waist index is a novel anthropometric index that incorporates both body fat distribution and weight, and has shown promise as a prognostic marker for adverse outcomes. Previous studies have demonstrated its utility in predicting mortality risk. For instance, in a prospective cohort of over 12,000 participants from southern China, Ding et al. reported a non-linear positive association between WWI and both all-cause and CVD mortality, with a significantly increased risk observed when WWI exceeded 11.2 cm/√kg (37). Similarly, Li et al. found a threshold effect in the association between WWI and CKD with a threshold of 9.8 cm/√kg (19). Similar to these findings, this study also identified a clear threshold effect. When WWI exceeded 10.5 cm/√kg, the risk of all outcomes, including all-cause mortality, CVD mortality and ESKD incidence, increased progressively with higher WWI levels, which was basically consistent with the correlation observed previously in the general population (20). In contrast, no significant associations were observed below this threshold, suggesting that WWI is only prognostically relevant beyond a certain level of central adiposity. This pattern contrasts with traditional obesity indicators such as BMI and WC, which often demonstrate J- or U-shaped relationships with adverse outcomes, reflecting potential adverse effects at both extremes (38, 39). Although widely used, BMI and WC have limited ability to distinguish between lean and fat mass (40). In contrast, previous studies have reported that elevated WWI values are associated with unfavorable body composition outcomes, including increased total and abdominal fat, reduced muscle mass, and decreased bone mass (17, 41). This distinctive association may partly account for WWI’s potential to mitigate the so-called “obesity paradox.” Research on sarcopenic obesity in CKD populations has shown that the coexistence of muscle wasting and excessive adiposity exacerbates metabolic dysregulation and increases vulnerability to frailty (42). In addition, central and visceral obesity have been identified as significant risk factors for both all-cause and CVD mortality in CKD patients, even among those with normal BMI or body weight (43). These observations suggest that anthropometric indices reflecting fat distribution may serve as superior prognostic tools in this population.

The lack of association in the lower WWI range may be attributable to its unique physiological implications. Lower WWI may reflect generally exhibit a more favorable body composition, characterized by reduced visceral fat and preserved skeletal muscle mass. Within this range, variability in anthropometric indices is more likely to reflect differences in lean tissue rather than metabolically deleterious fat accumulation (44). In addition, potential countervailing effects, such as the protective role of muscle reserves and nutritional status, may attenuate risk signals at lower WWI values. Nevertheless, the underlying biological mechanisms and their clinical relevance warrant further investigation, particularly to clarify whether this apparent risk attenuation is consistent across different populations and disease contexts.

The present findings align with prior research and further underscore the value of WWI as a clinically informative indicator, particularly among overweight individuals with central adiposity. By capturing the complex interplay between body fat distribution and body weight, WWI may provide a more precise assessment of obesity-related risk than conventional metrics. However, the underlying mechanisms linking WWI to adverse outcomes remain uncertain, particularly regarding the relative contributions of visceral adiposity, muscle mass, inflammation, and metabolic dysregulation. Furthermore, although WWI demonstrated a higher AUC than BMI and WC, the magnitude of improvement was modest. Therefore, the clinical implications of these findings should be interpreted cautiously, especially given the limited external validity of the study population. Based on the present findings, we suggest that WWI may be considered as a complementary indicator for risk stratification in clinical practice. WWI could be used alongside traditional measures such as BMI, WC, and laboratory indicators to provide a more comprehensive characterization of obesity-related risk. As a potentially modifiable anthropometric measure, WWI may also be explored as a target for lifestyle interventions; approaches aimed at reducing central adiposity through exercise and dietary modification could be hypothesized to confer renal and cardiovascular benefits in patients with CKD (45–47). Further prospective and interventional studies are required to substantiate the clinical utility of WWI and its role in guiding individualized management and preventive strategies.

This study presents several key strengths. First, it leverages a large, well-characterized UKB cohort, conferring substantial statistical power. Second, it applies rigorous analytical methods, including multivariate Cox proportional hazards models, competing risk models, RCS analysis and stepwise covariate adjustment, with WWI evaluated as both continuous and categorical variables to ensure analytical robustness. Third, the robustness of the findings was confirmed through sensitivity analysis excluding participants with major cardiovascular diseases, cancer at baseline, or less than 2 years of follow-up. The study also has limitations. Firstly, the study population was derived from the UK Biobank, which is predominantly composed of individuals of White European ancestry and underrepresents socioeconomically disadvantaged groups and body composition and anthropometric–outcome relationships vary across ethnic groups, the generalizability of these findings to broader and more diverse CKD populations may be limited. Extended follow-up may have introduced misclassification due to temporal changes in body composition or environmental exposures. CKD diagnosis and WWI were assessed based on baseline data, which precludes precise determination of the timing of disease onset or changes in WWI over time. Critically, detailed data on key medications, including renin–angiotensin–aldosterone system inhibitors, sodium–glucose cotransporter 2 inhibitors, and statins, were unavailable. Given the well-established effects of these therapies on metabolic profiles and cardiovascular–renal outcomes, their omission represents a major source of potential residual confounding and warrants explicit consideration when interpreting the results. Additionally, due to the observational design of the study, we were unable to explore the underlying biological mechanisms in depth. Nevertheless, these results offer valuable insights for comparable populations and highlight the need for validation in diverse cohorts, determination of optimal WWI thresholds, and prospective studies to evaluate its utility in guiding preventive and therapeutic strategies.

## 5 Conclusion

This study indicates that elevated WWI levels above the threshold are significantly associated with all-cause mortality, CVD mortality and the incidence of ESKD. Although WWI demonstrated statistically superior discriminatory performance compared with BMI and WC, the overall predictive ability was modest. Collectively, these findings suggest that WWI may serve as a complementary anthropometric indicator for risk assessment in CKD rather than a stand-alone predictive tool, and that further studies are warranted to clarify its role in clinical decision-making.

## Data Availability

The data sharing is not applicable to this article as UK Biobank data used was under a license and thus not publicly available. Access to the UK Biobank data can be requested through a standard protocol. Requests to access these datasets should be directed to https://www.ukbiobank.ac.uk/enable-your-research/apply-for-access.

## Footnotes

1
https://www.R-project.org/
