# Supplementary material for: Association between weight-adjusted waist index and risk of mortality and disease progression in participants with chronic kidney disease: a prospective study from the UK Biobank
Source: Front Nutr. 2026 Feb 25;13:1646414. doi: 10.3389/fnut.2026.1646414 (PMC12975434; doi:10.3389/fnut.2026.1646414)
Supplement: Supplementary file 1 [file Table_1.docx]

**Supplementary**

**Methods**

**Figure 1.** Cumulative incidence of all adverse outcomes in WWI quartile groups

**Figure 2.** RCS analysis of BMI with all adverse outcomes

**Figure 3.** RCS analysis of WC with all adverse outcomes

**Table 1.** The End-stage kidney disease related codes in the UK biobank

**Table 2.** Association of BMI with all-cause mortality, CVD mortality and ESKD incidence

**Table 3.** Association of WC with all-cause mortality, CVD mortality and ESKD incidence

**Table 4.** Subgroup analysis of the relationship between WWI and all-cause mortality, CVD mortality and ESKD incidence

**Table 5.** Association of WWI with all-cause mortality, CVD mortality and ESKD incidence excluding individuals with CVD or cancer at baseline

**Table 6.** Association of WWI with all-cause mortality, CVD mortality and ESKD incidence after excluding individuals experienced outcome events within the first two years of follow-up

**Table 7.** Association of WWI with CVD Mortality and ESKD Incidence Based on Cox Proportional Hazards Models with Competing Risks

**Methods**

*Definition of covariates*

Annual household income was separated into <18,000, 18,000–51,999, >52,000 or Other (“Do not know”, “Prefer not to answer”) in response to “What is the average total income before tax received by your HOUSEHOLD?” (Field 738).

Smoking status was categorized based on UK Biobank summary data as non-smoker (“Never”), ex-smoker (“Previous”), or current smoker (“Current”) base on the result of Field 20116.

Alcohol intake was separated into never (“Never”, “Special occasions only”), light (“One to three times a month”, “Once or twice a week”) or excessive (“Three or four times a week”, “Daily or almost daily”) in response to “About how often do you drink alcohol?” (Field 1558).

Education level was separated into college/above (“College or University degree”), high school/quivalent (“A levels/AS levels or equivalent”, “O levels/GCSEs or equivalent”), less than high school (“CSEs or equivalent”, “NVQ or HND or HNC or equivalent”, “Other professional qualifications eg: nursing, teaching”) or other (“None of the above”, “Prefer not to answer”) in response to “Which of the following qualifications do you have?” (Field 1558).

Physical activity was assessed using self-reported data from the UK Biobank baseline touchscreen questionnaire. Weekly minutes of moderate and vigorous physical activity were calculated by multiplying the reported frequency (Field 884 for moderate and Field 904 for vigorous activity) by the average duration per session (Field 894 and Field 914, respectively). Participants were classified as meeting the goal of American Heart Association (AHA) recommendations if they engaged in ≥150 minutes per week of moderate-intensity activity, ≥75 minutes per week of vigorous-intensity activity, or an equivalent combination.

Healthy diet score was assessed based on a touchscreen food frequency questionnaire (FFQ). Participants reported their daily intake of dietary consumption including poultry, beef, lamb/mutton, processed meat, oily fish, non-oily fish, fresh fruit, dried fruit, raw vegetables and cooked vegetables by answering touchscreen multiple-choice questions. Frequency categories of meat and fish were recoded: ‘never’ = 0, ‘less than once a week’ = 0.5, ‘once a week’ = 1, ‘2–4 times a week’ = 3, ‘5–6 times a week’ = 5.5 and ‘once or more daily’ = 7. Servings for beef, lamb/mutton and pork were summed to create the frequency of consumption of unprocessed red meat. For vegetables and fruit, participants were asked about how many heaped tablespoons of cooked/ salad or raw vegetables or pieces of fresh/dried fruit they consumed per day. Tablespoons of cooked/salad and raw vegetables were added to create the consumption of vegetables and pieces of fresh and dried fruit were added to create the consumption of fruit. We defined healthy diets according to the healthy diet score calculated on the basis of the following factors: vegetable intake of at least four tablespoons each day (median); fruit intake of at least three pieces each day (median); fish intake of at least twice each week (median); unprocessed red meat intake of no more than twice each week (median); and processed meat intake of no more than twice each week (median). One point was given for each favourable dietary factor and the total diet score ranged from 0 to 5. Participants were classified into three groups of poor (score of 0 or 1), medium (score of 2 or 3) or ideal (score of 4 or 5) (Field 1289-1349,Field 1369-1389).

Hypertension defined as having a mean systolic blood pressure (SBP) ≥ 140 mmHg or a mean diastolic blood pressure (DBP) ≥ 90 mmHg, a previous diagnosis by a physician, or self-reported use of antihypertensive medication within the past month.

Diabetes mellitus defined in accordance with the American Diabetes Association (ADA) criteria , which include meeting any one of the following conditions: (1) fasting blood glucose (FBG) ≥ 126 mg/dL (7.0 mmol/L); (2) 2-hour plasma glucose ≥ 200 mg/dL (11.1 mmol/L) during an oral glucose tolerance test (OGTT); (3) glycated hemoglobin (HbA1c) ≥ 6.5%; or (4) a documented history of diabetes diagnosis or use of glucose-lowering medications, including insulin or oral hypoglycemic agents.

Hyperuricemia characterized by an imbalance between uric acid (UA) production and excretion, defined as serum uric acid levels exceeding 7.0 mg/dL in men and 6.0 mg/dL in women.

**Figure 1. Cumulative incidence of all adverse outcomes in WWI quartile groups**

**
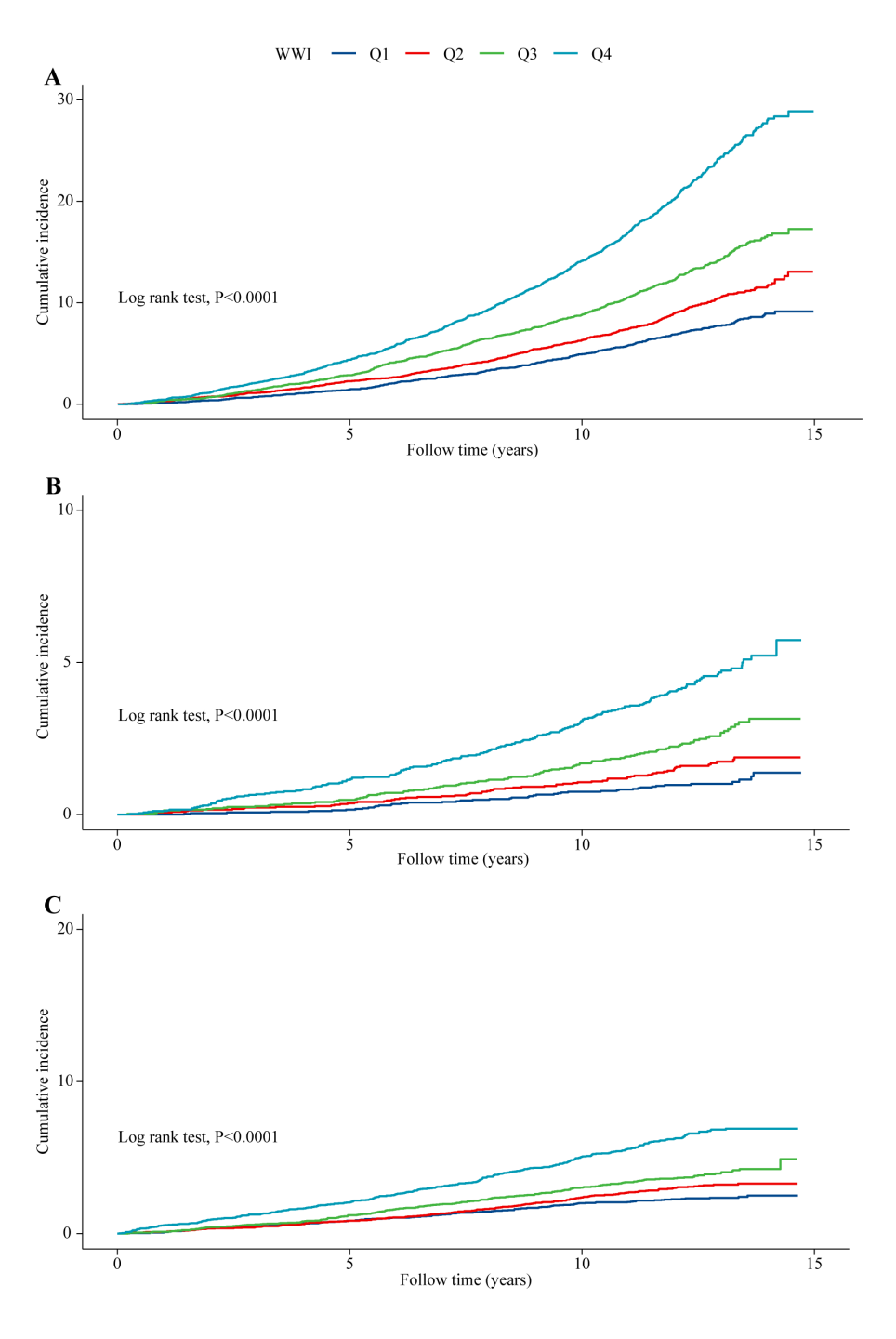
**

1. Cumulative all-cause mortality by WWI quartile groups. (B) Cumulative CVD mortality by WWI quartile groups.(C) Cumulative ESKD incidence by WWI quartile groups.

Abbreviations: WWI, weight-adjusted waist index

**Figure 2. RCS analysis of BMI with outcomes**


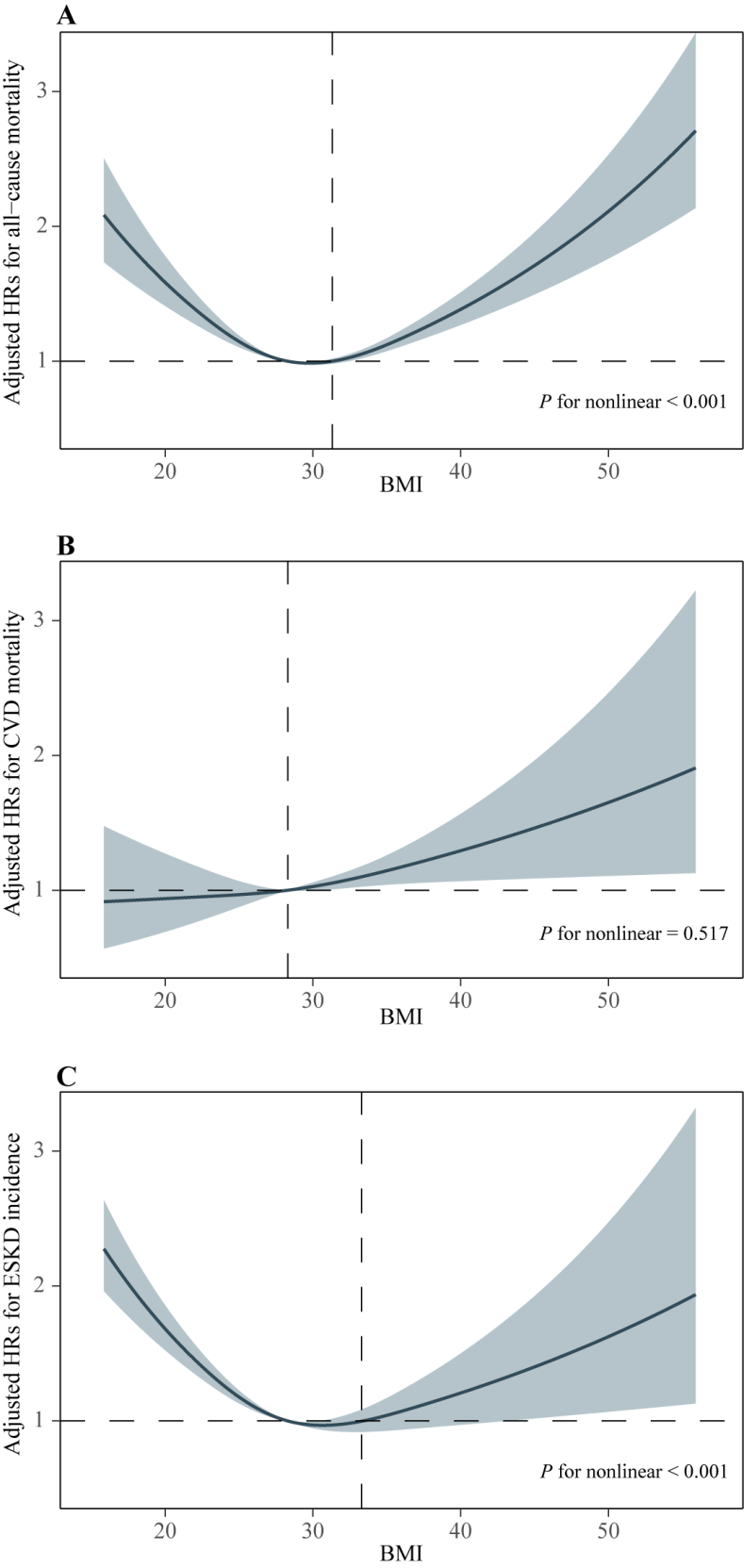


(A) all-cause mortality. (B) CVD mortality. (C) ESKD incidence.

Abbreviations: BMI, body mass index; CVD, cardiovascular; ESKD, end-stage kidney disease

**Figure 3. RCS analysis of WC with all adverse outcomes**

**
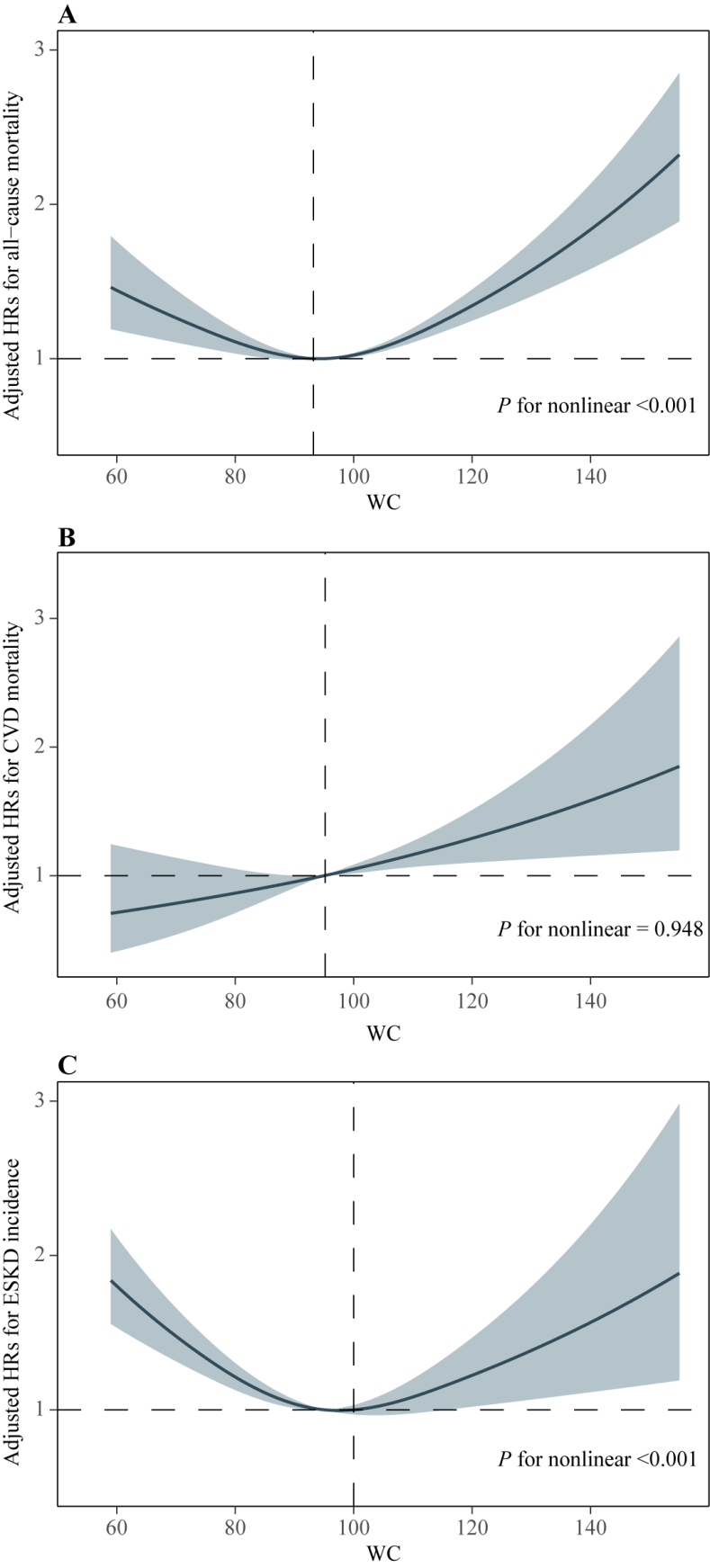
**

(A) all-cause mortality. (B) CVD mortality. (C) ESKD incidence

Abbreviations: WC, waist circumference; CVD, cardiovascular; ESKD, end-stage kidney disease

**Table 1. The End-stage kidney disease related codes in the UK biobank.**

| **Source** | **Code** | **Description** |
| --- | --- | --- |
| ICD10 | E85.3 | Secondary systemic amyloidosis |
|  | N16.5 | Renal tubulo-interstitial disorders in transplant rejection |
|  | N18.0 | Calculus of kidney with calculus of ureter |
|  | N18.5 | Urinary calculus, unspecified |
|  | Q60.1 | Unspecified renal colic |
|  | T82.4 | End-stage renal disease |
|  | T86.1 | Kidney transplant failure and rejection |
|  | Y60.2 | During kidney dialysis or other perfusion |
|  | Y61.2 | During kidney dialysis or other perfusion |
|  | Y62.2 | During kidney dialysis or other perfusion |
|  | Y84.1 | Kidney dialysis |
|  | Z49.0 | Preparatory care for dialysis |
|  | Z49.1 | Extracorporeal dialysis |
|  | Z49.2 | Other dialysis |
|  | Z94.0 | Kidney transplant status |
|  | Z99.2 | Dependence on renal dialysis |
| OPSC-4 | L74.1 | Insertion of arteriovenous prosthesis |
|  | L74.2 | Creation of arteriovenous fistula NEC |
|  | L74.3 | Attention to arteriovenous shunt |
|  | L74.4 | Banding of arteriovenous fistula |
|  | L74.5 | Thrombectomy of arteriovenous fistula |
|  | L74.6 | Creation of graft fistula for dialysis |
|  | L74.8 | Other specified arteriovenous shunt |
|  | L74.9 | Unspecified arteriovenous shunt |
|  | M01.2 | Allotransplantation of kidney from live donor |
|  | M01.3 | Allotransplantation of kidney from cadaver NEC |
|  | M01.4 | Allotransplantation of kidney from cadaver heart beating |
|  | M01.5 | Allotransplantation of kidney from cadaver heart non-beating |
|  | M01.8 | Other specified transplantation of kidney |
|  | M01.9 | Unspecified transplantation of kidney |
|  | M02.3 | Bilateral nephrectomy |
|  | M08.4 | Exploration of transplanted kidney |
|  | M17.2 | Pre-transplantation of kidney work-up - recipient |
|  | M17.4 | Post-transplantation of kidney examination - recipient |
|  | M17.8 | Other specified interventions associated with transplantation of kidney |
|  | M17.9 | Unspecified interventions associated with transplantation of kidney |
|  | X40.1 | Renal dialysis |
|  | X40.2 | Peritoneal dialysis NEC |
|  | X40.3 | Haemodialysis NEC |
|  | X40.4 | Haemofiltration |
|  | X40.5 | Automated peritoneal dialysis |
|  | X40.6 | Continuous ambulatory peritoneal dialysis |
|  | X40.7 | Haemoperfusion |
|  | X40.8 | Other specified compensation for renal failure |
|  | X40.9 | Unspecified compensation for renal failure |
|  | X41.1 | Insertion of ambulatory peritoneal dialysis catheter |
|  | X41.2 | Removal of ambulatory peritoneal dialysis catheter |
|  | X41.8 | Other specified placement of ambulatory apparatus for compensation for renal failure |
|  | X41.9 | Unspecified placement of ambulatory apparatus for compensation for renal failure |
|  | X42.1 | Insertion of temporary peritoneal dialysis catheter |
|  | X42.8 | Other specified placement of other apparatus for compensation for renal failure |
|  | X42.9 | Unspecified placement of other apparatus for compensation for renal failure |
|  | X43.1 | Extracorporeal albumin haemodialysis |
| Death register | N18.0 | End-stage renal disease |
|  | N18.5 | Chronic kidney disease, stage 5 |

**Table 2. Association of BMI with all-cause mortality, CVD mortality and ESKD incidence.**

|  |  | **Hazard ratio (95% CI)**^a^ | | |
| --- | --- | --- | --- | --- |
| **BMI** | **Cases/N** | **Model 1** | **Model 2** | **Model 3** |
| **All-cause** |  |  |  |  |
| All-cause mortality | 3,874/22,523 | 1.03 (1.02-1.03)^***^ | 1.00 (1.00-1.01) | 1.00 (1.00-1.01)^*^ |
| BMI (Per-1 increase) |  |  |  |  |
| BMI (quartile) |  |  |  |  |
| Quartile 1 | 794/5,373 | 1 (ref) | 1 (ref) | 1 (ref) |
| Quartile 2 | 862/5,659 | 0.82 (0.75-0.91)^***^ | 0.81 (0.74-0.90)^***^ | 0.80 (0.72-0.90)^***^ |
| Quartile 3 | 999/5,687 | 0.92 (0.83-1.01) | 0.82 (0.74-0.90)^***^ | 0.82 (0.74-0.92)^***^ |
| Quartile 4 | 1,219/5,804 | 1.27 (1.16-1.39)^***^ | 0.93 (0.84-1.03) | 0.97 (0.87-1.08) |
| *P* for trend |  | <0.001 | >0.05 | >0.05 |
| CVD cause mortality |  |  |  |  |
| BMI (Per-1 increase) | 786/22,523 | 1.05 (1.04-1.06)^***^ | 1.01 (0.99-1.02) | 1.01 (1.00-1.03) |
| BMI (quartile) |  |  |  |  |
| Quartile 1 | 108/5,373 | 1 (ref) | 1 (ref) | 1 (ref) |
| Quartile 2 | 170/5,659 | 1.11 (0.87-1.42) | 1.03 (0.80-1.31) | 0.97 (0.74-1.27) |
| Quartile 3 | 227/5,687 | 1.39 (1.10-1.75)^**^ | 1.08 (0.85-1.38) | 1.06 (0.82-1.37) |
| Quartile 4 | 281/5,804 | 1.98 (1.59-2.48)^***^ | 1.14 (0.89-1.46) | 1.16 (0.90-1.51) |
| *P* for trend |  | <0.001 | >0.05 | >0.05 |
| ESKD incidence |  |  |  |  |
| BMI (Per-1 increase) | 823/22,523 | 1.04 (1.02-1.05)^***^ | 0.98 (0.97-0.99)^**^ | 0.99 (0.98-1.01) |
| BMI (quartile) |  |  |  |  |
| Quartile 1 | 157/5,373 | 1 (ref) | 1 (ref) | 1 (ref) |
| Quartile 2 | 168/5,659 | 0.85 (0.68-1.06) | 0.67 (0.54-0.84)^***^ | 0.62 (0.48-0.79)^***^ |
| Quartile 3 | 223/5,687 | 1.08 (0.88-1.33) | 0.67 (0.54-0.83)^***^ | 0.69 (0.54-0.86)^**^ |
| Quartile 4 | 275/5,804 | 1.42 (1.16-1.73)^***^ | 0.62 (0.49-0.77)^***^ | 0.68 (0.54-0.86)^**^ |
| *P* for trend |  | <0.001 | <0.001 | <0.05 |

Abbreviations: BMI, body mass index; CVD, cardiovascular disease; ESKD, end-stage kidney disease

^a^ Model 1: Age and sex were adjusted. Model 2: Age, sex, annual household income, smoking status, alcohol intake, education level, townsend deprivation index, healthy diet score, physical activity, hyperuricemia, hypertension, diabetes mellitus, HDLC, LDLC, Triglycerides and WBC were adjusted. Model 3: Age, sex, annual household income, smoking status, alcohol intake, education level, townsend deprivation index, healthy diet score, physical activity, hyperuricemia, hypertension, diabetes mellitus, HDLC, LDLC, Triglycerides, WBC, eGFR and UACR were adjusted

**Table 3. Association of WC with all-cause mortality, CVD mortality and ESKD incidence.**

|  |  | **Hazard ratio (95% CI)**^a^ | | |
| --- | --- | --- | --- | --- |
| **WC** | **Cases/N** | **Model 1** | **Model 2** | **Model 3** |
| All-cause mortality |  |  |  |  |
| WC (Per-1 increase) | 3,874/22,523 | 1.02 (1.01-1.02)^***^ | 1.01 (1.00-1.01)^***^ | 1.01 (1.00-1.01)^***^ |
| WC (quartile) |  |  |  |  |
| Quartile 1 | 585/5,338 | 1 (ref) | 1 (ref) | 1 (ref) |
| Quartile 2 | 809/5,730 | 0.94 (0.84-1.05) | 0.86 (0.77-0.96)^**^ | 0.84 (0.74-0.95)^**^ |
| Quartile 3 | 1,013/5,595 | 1.09 (0.97-1.22) | 0.91 (0.81-1.02) | 0.93 (0.82-1.05) |
| Quartile 4 | 1,467/5,860 | 1.54 (1.38-1.71)^***^ | 1.10 (0.97-1.24) | 1.12 (0.98-1.27) |
| *P* for trend |  | <0.001 | <0.001 | <0.001 |
| CVD cause mortality |  |  |  |  |
| WC (Per-1 increase) | 786/22,523 | 1.02 (1.02-1.03)^***^ | 1.01 (1.00-1.01)^*^ | 1.01 (1.00-1.01)^**^ |
| WC (quartile) |  |  |  |  |
| Quartile 1 | 68/5,338 | 1 (ref) | 1 (ref) | 1 (ref) |
| Quartile 2 | 132/5,730 | 1.14 (0.84-1.55) | 0.91 (0.67-1.24) | 0.81 (0.58-1.13) |
| Quartile 3 | 243/5,595 | 1.82 (1.36-2.43)^***^ | 1.23 (0.91-1.66) | 1.22 (0.89-1.67) |
| Quartile 4 | 343/5,860 | 2.43 (1.83-3.23)^***^ | 1.26 (0.92-1.70) | 1.21 (0.88-1.68) |
| *P* for trend |  | <0.001 | <0.05 | <0.05 |
| ESKD incidence |  |  |  |  |
| WC (Per-1 increase) | 823/22,523 | 1.02 (1.01-1.02)^***^ | 1.00 (0.99-1.00) | 1.00 (0.99-1.01) |
| WC (quartile) |  |  |  |  |
| Quartile 1 | 118/5,338 | 1 (ref) | 1 (ref) |  |
| Quartile 2 | 160/5,730 | 0.99 (0.77-1.27) | 0.69 (0.54-0.89)^**^ | 0.64 (0.48-0.84)^**^ |
| Quartile 3 | 228/5,595 | 1.30 (1.02-1.65)^*^ | 0.71 (0.56-0.92)^**^ | 0.80 (0.61-1.04) |
| Quartile 4 | 317/5,860 | 1.71 (1.35-2.17)^***^ | 0.69 (0.53-0.89)^**^ | 0.83 (0.63-1.09) |
| *P* for trend |  | <0.001 | >0.05 | >0.05 |

Abbreviations: WC, waist Circumference; CVD, cardiovascular disease; ESKD, end-stage kidney disease

a Model 1: Age and sex were adjusted. Model 2: Age, sex, annual household income, smoking status, alcohol intake, education level, townsend deprivation index, healthy diet score, physical activity, hyperuricemia, hypertension, diabetes mellitus, HDLC, LDLC, Triglycerides and WBC were adjusted. Model 3: Age, sex, annual household income, smoking status, alcohol intake, education level, townsend deprivation index, healthy diet score, physical activity, hyperuricemia, hypertension, diabetes mellitus, HDLC, LDLC, Triglycerides, WBC, eGFR and UACR were adjusted

**Table 4. Subgroup analysis of the relationship between WWI and all-cause mortality, CVD mortality and ESKD incidence.**

|  | N | **All-cause mortality** | | | **CVD mortality** | | | **ESKD incidence** | | |
| --- | --- | --- | --- | --- | --- | --- | --- | --- | --- | --- |
|  |  | cases | HR (95%CI) | *P* for  interaction | cases | HR (95%CI) | *P* for  interaction | cases | HR (95%CI) | *P* for  interaction |
| Sex |  |  |  | 0.072 |  |  | 0.022 |  |  | 0.621 |
| Male | 11,046 | 2,540 | 1.26 (1.18-1.34) |  | 601 | 1.15 (1.01-1.30) |  | 556 | 1.13 (0.99-1.29) |  |
| Female | 11,477 | 1,334 | 1.20 (1.11-1.28) |  | 185 | 1.50 (1.24-1.81) |  | 267 | 1.15 (0.98-1.35) |  |
| Age, years |  |  |  | <0.001 |  |  | 0.03 |  |  | 0.056 |
| <60 | 9,084 | 793 | 1.43 (1.29-1.58) |  | 154 | 1.38 (1.09-1.74) |  | 295 | 1.04 (0.88-1.22) |  |
| ≥60 | 13,439 | 3,081 | 1.22 (1.16-1.28) |  | 632 | 1.25 (1.11,1.40) |  | 528 | 1.24 (1.09-1.41) |  |
| Hypertension |  |  |  | 0.016 |  |  | 0.024 |  |  | 0.249 |
| Yes | 17,958 | 3,191 | 1.26 (1.19-1.32) |  | 658 | 1.27 (1.13-1.42) |  | 680 | 1.15 (1.03-1.28) |  |
| No | 4,565 | 683 | 1.13 (1.01-1.26) |  | 128 | 1.16 (0.90-1.50) |  | 143 | 1.08 (0.84-1.38) |  |
| Hyperuricemia |  |  |  | 0.039 |  |  | 0.011 |  |  | <0.001 |
| Yes | 6,782 | 1,553 | 1.24 (1.15-1.34) |  | 370 | 1.18 (1.01-1.37) |  | 525 | 1.04 (0.92-1.18) |  |
| No | 15,741 | 2,321 | 1.23 (1.16-1.31) |  | 416 | 1.32 (1.14-1.52) |  | 298 | 1.26 (1.07-1.50) |  |
| Diabetes |  |  |  | 0.591 |  |  | 0.266 |  |  | 0.58 |
| Yes | 6,813 | 1,585 | 1.32 (1.23-1.42) |  | 395 | 1.26 (1.08-1.46) |  | 360 | 1.23 (1.06-1.44) |  |
| No | 5,710 | 2,289 | 1.17 (1.10-1.24) |  | 391 | 1.24 (1.07-1.45) |  | 463 | 1.05 (0.92-1.21) |  |

Abbreviations: WWI, weight-adjusted waist index; CVD, cardiovascular disease; ESKD, end-stage kidney disease

**Table 5. Association of WWI with all-cause mortality, CVD mortality and ESKD incidence excluding individuals with CVD or cancer at baseline.**

|  |  | **Hazard ratio (95% CI)**^a^ | | |
| --- | --- | --- | --- | --- |
| **WWI** | **Cases/N** | **Model 1** | **Model 2** | **Model 3** |
| All-cause mortality |  |  |  |  |
| WWI (Per-1 increase) | 2,335/17,564 | 1.49 (1.41-1.57)^***^ | 1.30 (1.22-1.38)^***^ | 1.31 (1.23-1.39)^***^ |
| WWI (quartile) |  |  |  |  |
| Quartile 1 | 356/4,665 | 1 (ref) | 1 (ref) | 1 (ref) |
| Quartile 2 | 478/4,523 | 1.08 (0.94-1.24) | 0.99 (0.86-1.14) | 1.03 (0.89-1.20) |
| Quartile 3 | 595/4,330 | 1.27 (1.11-1.45)^***^ | 1.05 (0.91-1.21) | 1.11 (0.95-1.29) |
| Quartile 4 | 906/4,046 | 2.00 (1.76-2.27)^***^ | 1.42 (1.23-1.63)^***^ | 1.45 (1.25-1.69)^***^ |
| *P* for trend |  | <0.001 | <0.001 | <0.001 |
| CVD cause mortality |  |  |  |  |
| WWI (Per-1 increase) | 407/17,564 | 1.63 (1.43-1.86)^***^ | 1.28 (1.11-1.48)^***^ | 1.25 (1.08-1.46)^**^ |
| WWI (quartile) |  |  |  |  |
| Quartile 1 | 48/4,665 | 1 (ref) | 1 (ref) | 1 (ref) |
| Quartile 2 | 75/4,523 | 1.13 (0.78-1.64) | 0.95 (0.66-1.38) | 0.99 (0.67-1.47) |
| Quartile 3 | 112/4,330 | 1.54 (1.08-2.18)^*^ | 1.12 (0.78-1.60) | 1.14 (0.78-1.66) |
| Quartile 4 | 172/4,046 | 2.41 (1.73-3.37)^***^ | 1.42 (1.00-2.03) | 1.40 (0.96-2.03) |
| *P* for trend |  | <0.001 | <0.01 | <0.05 |
| ESKD incidence |  |  |  |  |
| WWI (Per-1 increase) | 559/17,564 | 1.56 (1.40-1.74)^***^ | 1.18 (1.05-1.33)^**^ | 1.22 (1.07-1.40)^**^ |
| WWI (quartile) |  |  |  |  |
| Quartile 1 | 127/5,593 | 1 (ref) | 1 (ref) | 1 (ref) |
| Quartile 2 | 166/5,640 | 1.09 (0.82-1.43) | 0.88 (0.66-1.16) | 1.10 (0.81-1.49) |
| Quartile 3 | 205/5,656 | 1.17 (0.89-1.55) | 0.80 (0.60-1.07) | 1.08 (0.79-1.47) |
| Quartile 4 | 325/5,634 | 2.14 (1.66-2.78)^***^ | 1.17 (0.89-1.55) | 1.34 (0.98-1.84) |
| *P* for trend |  | <0.001 | >0.05 | >0.05 |

Abbreviations: WWI, weight-adjusted waist index; CVD, cardiovascular disease; ESKD, end-stage kidney disease

a Model 1: Age and sex were adjusted. Model 2: Age, sex, annual household income, smoking status, alcohol intake, education level, townsend deprivation index, healthy diet score, physical activity, hyperuricemia, hypertension, diabetes mellitus, HDLC, LDLC, Triglycerides and WBC were adjusted. Model 3: Age, sex, annual household income, smoking status, alcohol intake, education level, townsend deprivation index, healthy diet score, physical activity, hyperuricemia, hypertension, diabetes mellitus, HDLC, LDLC, Triglycerides, WBC, eGFR and UACR were adjusted

**Table 6. Association of WWI with all-cause mortality, CVD mortality and ESKD incidence after excluding individuals experienced outcome events within the first two years of follow-up**

|  |  | **Hazard ratio (95% CI)**^a^ | | |
| --- | --- | --- | --- | --- |
| **WWI** | **Cases/N** | **Model 1** | **Model 2** | **Model 3** |
| All-cause mortality |  |  |  |  |
| WWI (Per-1 increase) | 3,490/22,095 | 1.47 (1.41-1.54)^***^ | 1.27 (1.21-1.34)^***^ | 1.30 (1.24-1.37)^***^ |
| WWI (quartile) |  |  |  |  |
| Quartile 1 | 487/5,537 | 1 (ref) | 1 (ref) | 1 (ref) |
| Quartile 2 | 701/5,549 | 1.12 (0.99-1.26) | 1.01 (0.89-1.13) | 1.05 (0.92-1.20) |
| Quartile 3 | 912/5,550 | 1.31 (1.17-1.47)^***^ | 1.06 (0.95-1.20) | 1.13 (1.00-1.29) |
| Quartile 4 | 1390/5,459 | 1.99 (1.78-2.21)^***^ | 1.38 (1.23-1.55)^***^ | 1.45 (1.28-1.65)^***^ |
| *P* for trend |  | <0.001 | <0.001 | <0.001 |
| CVD cause mortality |  |  |  |  |
| WWI (Per-1 increase) | 685/22,095 | 1.71 (1.54-1.90)^***^ | 1.29 (1.15-1.44)^***^ | 1.29 (1.14-1.46)^***^ |
| WWI (quartile) |  |  |  |  |
| Quartile 1 | 70/5,537 | 1 (ref) | 1 (ref) | 1 (ref) |
| Quartile 2 | 119/5,549 | 1.16 (0.86-1.56) | 0.94 (0.69-1.27) | 0.98 (0.71-1.36) |
| Quartile 3 | 178/5,550 | 1.49 (1.12-1.99)^**^ | 1.03 (0.77-1.38) | 1.04 (0.76-1.43) |
| Quartile 4 | 318/5,459 | 2.62 (2.00-3.43)^***^ | 1.43 (1.07-1.90)^*^ | 1.45 (1.07-1.97)^*^ |
| *P* for trend |  | <0.001 | <0.001 | <0.001 |
| ESKD incidence |  |  |  |  |
| WWI (Per-1 increase) | 709/22,095 | 1.53 (1.38-1.68)^***^ | 1.14 (1.02-1.27)^*^ | 1.26 (1.12-1.42)^***^ |
| WWI (quartile) |  |  |  |  |
| Quartile 1 | 107/5,537 | 1 (ref) | 1 (ref) | 1 (ref) |
| Quartile 2 | 147/5,549 | 1.12 (0.87-1.45) | 0.87 (0.67-1.13) | 1.11 (0.84-1.47) |
| Quartile 3 | 181/5,550 | 1.31 (1.02-1.69) | 0.87 (0.68-1.13) | 1.12 (0.85-1.49) |
| Quartile 4 | 274/5,459 | 2.07 (1.64-2.63)^***^ | 1.09 (0.85-1.41) | 1.38 (1.04-1.82)^*^ |
| *P* for trend |  | <0.001 | >0.05 | <0.05 |

Abbreviations: WWI, weight-adjusted waist index; CVD, cardiovascular disease; ESKD, end-stage kidney disease

a Model 1: Age and sex were adjusted. Model 2: Age, sex, annual household income, smoking status, alcohol intake, education level, townsend deprivation index, healthy diet score, physical activity, hyperuricemia, hypertension, diabetes mellitus, HDLC, LDLC, Triglycerides and WBC were adjusted. Model 3: Age, sex, annual household income, smoking status, alcohol intake, education level, townsend deprivation index, healthy diet score, physical activity, hyperuricemia, hypertension, diabetes mellitus, HDLC, LDLC, Triglycerides, WBC, eGFR and UACR were adjusted

**Table 7. Association of WWI with CVD Mortality and ESKD Incidence Based on Cox Proportional Hazards Models with Competing Risks**

|  | **Hazard ratio (95% CI)**^a^ | | |
| --- | --- | --- | --- |
| **WWI** | **Model 1** | **Model 2** | **Model 3** |
| CVD cause mortality |  |  |  |
| WWI (Per-1 increase) | 1.61 (1.44-1.79)^***^ | 1.21 (1.08-1.35)^**^ | 1.21 (1.08-1.36)^**^ |
| WWI (quartile) |  |  |  |
| Quartile 1 | 1 (ref) | 1 (ref) | 1 (ref) |
| Quartile 2 | 1.26 (0.93-1.72) | 1.03 (0.76-1.40) | 1.04 (0.77-1.42) |
| Quartile 3 | 1.54 (1.15-2.07)^**^ | 1.06 (0.78-1.42) | 1.06 (0.78-1.43) |
| Quartile 4 | 2.52 (1.89-3.36)^***^ | 1.37 (1.02-1.84)^*^ | 1.39 (1.03-1.87)^*^ |
| *P* for trend | <0.001 | <0.01 | <0.01 |
| ESKD incidence |  |  |  |
| WWI (Per-1 increase) | 1.44 (1.30-1.58)^***^ | 1.08 (0.97-1.20)^*^ | 1.19 (1.06-1.34)^**^ |
| WWI (quartile) |  |  |  |
| Quartile 1 | 1 (ref) | 1 (ref) | 1 (ref) |
| Quartile 2 | 1.12 (0.87-1.46) | 0.88 (0.68-1.14) | 1.02 (0.79-1.32) |
| Quartile 3 | 1.24 (0.96-1.59) | 0.83 (0.64-1.08) | 1.04 (0.80-1.36) |
| Quartile 4 | 1.92 (1.51-2.45)^***^ | 1.03 (0.79-1.33) | 1.34 (1.03-1.75)^*^ |
| *P* for trend | <0.001 | >0.05 | <0.05 |

Abbreviations: WWI, weight-adjusted waist index; CVD, cardiovascular disease; ESKD, end-stage kidney disease

a Model 1: Age and sex were adjusted. Model 2: Age, sex, annual household income, smoking status, alcohol intake, education level, townsend deprivation index, healthy diet score, physical activity, hyperuricemia, hypertension, diabetes mellitus, HDLC, LDLC, Triglycerides and WBC were adjusted. Model 3: Age, sex, annual household income, smoking status, alcohol intake, education level, townsend deprivation index, healthy diet score, physical activity, hyperuricemia, hypertension, diabetes mellitus, HDLC, LDLC, Triglycerides, WBC, eGFR and UACR were adjusted

**Table 8. Pairwise Comparison of Predictive Performance (AUC) Among WWI, BMI, and WC for Adverse Outcomes**

| **Outcome** | **Comparison** | ***P*-value (bootstrap)** | **Adjusted *P*-value (Bonferroni)** |
| --- | --- | --- | --- |
| All-cause mortality | WWI vs. BMI | < 0.001 | < 0.001 |
|  | WWI vs. WC | < 0.001 | < 0.001 |
|  | BMI vs. WC | < 0.001 | < 0.001 |
| CVD mortality | WWI vs. BMI | < 0.001 | < 0.001 |
|  | WWI vs. WC | 0.019 | 0.057 |
|  | BMI vs. WC | < 0.001 | < 0.001 |
| ESKD incidence | WWI vs. BMI | < 0.001 | < 0.001 |
|  | WWI vs. WC | 0.272 | 0.816 |
|  | BMI vs. WC | < 0.001 | < 0.001 |

Abbreviations: WWI, weight-adjusted waist index; eGFR, estimated glomerular filtration rate; UACR, urinary albumin-to-creatinine ratio; CVD, cardiovascular disease; ESKD, end-stage kidney disease
